# Brief research report: impact of vaccination on antibody responses and mortality from severe COVID-19

**DOI:** 10.3389/fimmu.2024.1325243

**Published:** 2024-02-07

**Authors:** Bindu Adhikari, Joseph S. Bednash, Jeffrey C. Horowitz, Mark P. Rubinstein, Anastasia N. Vlasova

**Affiliations:** ^1^ Department of Veterinary Preventive Medicine, College of Veterinary Medicine, The Ohio State University, Wooster, OH, United States; ^2^ Center for Food Animal Health, Department of Animal Sciences, Ohio Agriculture Research and Development Center (OARDC), College of Food, Agricultural and Environmental Sciences, The Ohio State University, Wooster, OH, United States; ^3^ Department of Internal Medicine, Division of Pulmonary, Critical Care, and Sleep Medicine, The Ohio State University, Columbus, OH, United States; ^4^ Division of Medical Oncology, Department of Internal Medicine, The Ohio State University, Columbus, OH, United States; ^5^ The Pelotonia Institute of Immuno-Oncology, The Ohio State University James Comprehensive Cancer Center, Columbus, OH, United States

**Keywords:** SARS-CoV-2, mortality, antibodies, IgG4, immune tolerance, comorbidities, COVID-19 vaccines

## Abstract

**Introduction:**

While it is established that vaccination reduces risk of hospitalization, there is conflicting data on whether it improves outcome among hospitalized COVID-19 patients. This study evaluated clinical outcomes and antibody (Ab) responses to severe acute respiratory syndrome coronavirus-2 (SARS-CoV-2) infection/vaccines in patients with acute respiratory failure (ARF) and various comorbidities.

**Methods:**

In this single-center study, 152 adult patients were admitted to Ohio State University hospital with ARF (05/2020 – 11/2022) including 112 COVID-19-positive and 40 COVID-19-negative patients. Of the COVID-19 positive patients, 23 were vaccinated for SARS-CoV-2 (Vax), and 89 were not (NVax). Of the NVax COVID-19 patients, 46 were admitted before and 43 after SARS-CoV-2 vaccines were approved. SARS-CoV-2 Ab levels were measured/analyzed based on various demographic and clinical parameters of COVID-19 patients. Additionally, total IgG4 Ab concentrations were compared between the Vax and NVax patients.

**Results:**

While mortality rates were 36% (n=25) and 27% (n=15) for non-COVID-19 NVax and Vax patients, respectively, in COVID-19 patients mortality rates were 37% (NVax, n=89) and 70% (Vax, n=23). Among COVID-19 patients, mortality rate was significantly higher among Vax vs. NVax patients (p=0.002). The Charlson’s Comorbidity Index score (CCI) was also significantly higher among Vax vs. NVax COVID-19 patients. However, the mortality risk remained significantly higher (p=0.02) when we compared COVID-19 Vax vs. NVax patients with similar CCI score, suggesting that additional factors may increase risk of mortality. Higher levels of SARS-CoV-2 Abs were noted among survivors, suggestive of their protective role. We observed a trend for increased total IgG4 Ab, which promotes immune tolerance, in the Vax vs. NVax patients in week 3.

**Conclusion:**

Although our cohort size is small, our results suggest that vaccination status of hospital-admitted COVID-19 patients may not be instructive in determining mortality risk. This may reflect that within the general population, those individuals at highest risk for COVID-19 mortality/immune failure are likely to be vaccinated. Importantly, the value of vaccination may be in preventing hospitalization as opposed to stratifying outcome among hospitalized patients, although our data do not address this possibility. Additional research to identify factors predictive of aberrant immunogenic responses to vaccination is warranted.

## Introduction

The coronavirus disease 2019 (COVID-19) pandemic has presented numerous challenges and health threats, particularly for individuals with comorbidities (1). Vaccination against COVID-19 has conferred widespread protection against serious illness, with hospitalization rates significantly reduced for fully vaccinated people in all demographics studied (2, 3). Initially, vaccines were prioritized for high-risk populations (elderly and those with comorbidities); however, emerging evidence suggests that vaccination may not benefit or may even represent an additional risk for subsets of patients, highlighting the need for further investigation (4, 5). Here, we studied the impact of COVID-19 mRNA vaccines on protection against COVID-19-associated acute respiratory failure (ARF) in the context of SARS-CoV-2 antibody (Ab) responses. Additionally, we evaluated whether preexisting common cold coronavirus (CCCoV) Ab could alter COVID-19 pathogenesis.

## Materials and methods

We obtained banked plasma samples from the Ohio State University Intensive Care Unit Registry (BuckICU) collected from individuals admitted to The Ohio State University (OSU) hospitals from May 2020 to November 2022. This biorepository collects longitudinal biospecimens and associated clinical data from hospitalized patients tested positive for COVID-19 (by RT-PCR) and non-COVID-19 respiratory failure of varying severity. Notably, the cohort is enriched for critically ill patients admitted to the Intensive Care Unit (ICU). Plasma collection, enzyme-linked immunosorbent assay (ELISA) development and validation, and ELISA protocols are detailed in our recent study (6).

**Figure 1:**
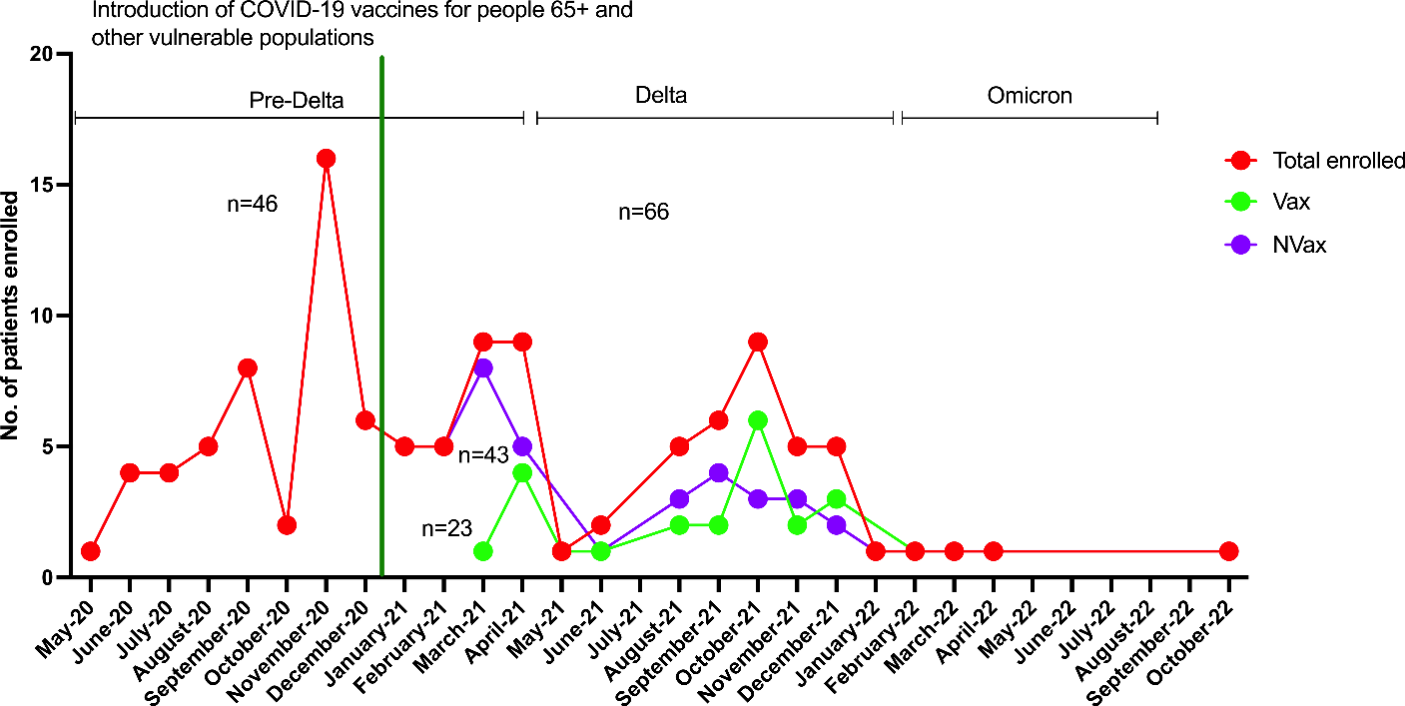
Numbers of Vax and NVax SARS-CoV-2 positive patients enrolled between May 2020 and November 2022, before (May 2020 – mid-December 2020) and after (after mid-December 2020) vaccine roll-out. The green vertical line indicates the time of vaccine use authorization for 65+/vulnerable populations (December 17^th^, 2020).

A total of 152 adult (18+ years) patients of both sexes were enrolled including SARS-CoV-2 infected (n=112, 74%) and non-infected (n=40, 26%) patients. Out of the 40 non-COVID patients, a total of 15 (38%) patients were vaccinated (Vax), 25 (62%) were not vaccinated (NVax). Out of 112 SARS-CoV-2 infected patients, 23 patients (20%) were vaccinated against SARS-CoV-2 (Vax), while the remaining 89 individuals (80%) were not (NVax). Out of the 23 Vax patients, 26% (6 patients) had received 3 vaccine doses, 30% (7 patients) had received 2 doses (BNT162b2/mRNA-1273), 5% (1 patient) had received 2 doses of an unspecified (SARS-CoV-2) vaccine, and 9% (2 patients) received Ad26.COV2-S vaccine at least two weeks prior to hospital admission. Thirty percent (7 patients) had received incomplete vaccine series before the hospital admission. Thus, most patients in this cohort received mRNA vaccines. Comparative data on demographic/clinical variables are detailed in **Supplementary Table 1**. Forty-six COVID-19 patients were admitted prior to vaccine introduction, while following the vaccine roll-out 23 Vax and 43 NVax COVID-19 patients were admitted (**Figure 1**).

Charlson’s Comorbidity Index score (CCI, the score calculated from a weighted index consisted of age and the number/seriousness of comorbid diseases) was used to compare Vax vs. NVax patients. Using previously established ELISA protocols (6), we measured CCCoV/SARS-CoV-2 Ab levels in the plasma of this cohort. Also, we used IgG4 human ELISA (Invitrogen, catalog #BMS2095) following the manufacturer’s instruction and compared total IgG4 Ab concentrations among the Vax (n=20) and NVax (n=20) patients at three different time points (week 1, week 2 and week 3).

Most of the statistical analyses were performed using PRISM 10 (GraphPad).For the statistical analysis Kaplan Meier survival analysis was conducted using R studio. Mann-Whitney and Kruskal–Wallis *post hoc* test was used to compare unpaired values. Fisher’s exact test was used for the **Supplementary Table 1** data. The significance level of 0.05 was used to determine significance; *p < 0.05 and ***p < 0.001.

## Results

**Figure 2:**
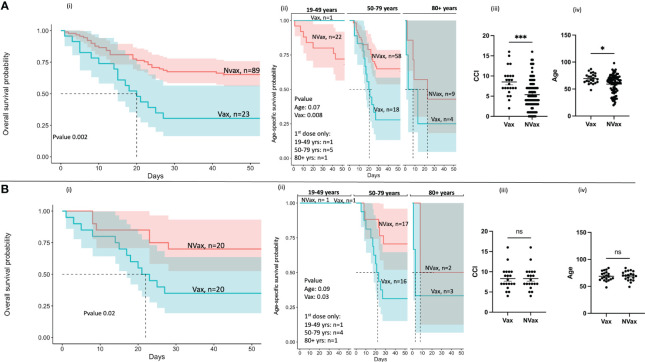
Kaplan-Meier survival analysis of SARS-CoV-2 infected patients before **(A)** and after **(B)** adjustment for Charlson’s Comorbidity Index (CCI). (i) Overall survival probability of the Vax and NVax SARS-CoV-2-infected patients (ii) Age-specific survival probability of the Vax and NVax patients from the three age groups (19-49 years, 50-79 years, and 80+ years), (iii) Scatter plots (median, 95%CI) showing CCI and **(iv)** age of the Vax vs. NVax patients. The dotted lines on the survival plots represent the median survival time. Shaded area represents a 95% confidence interval. Differences were considered significant at a p-value < 0.05(*), and <0.001(***). ns, non-significant.

In our cohort of COVID-19 patients admitted to the hospital between May 2020 and November 2022, we accrued 112 individuals, including Vax 23 and 89 NVax individuals. Of the 66 patients admitted to the hospital with severe COVID-19 between December 2020 and November 2022 (after vaccines became available), 23 were Vax and 43 were NVax (**Figure 1**). Interestingly, mortality among Vax patients in this cohort was 70% compared with 37% in the NVax group, and overall survival rate was ~2 times higher in the NVax patients (p=0.0086, **Supplementary Table 1**). Because there was only 1 Vax COVID-19 patient in the youngest age group (19-49 years), it precluded comparison of survival rates in this age group. Of interest, NVax patients aged 19-49 and 50-79 years had somewhat similar survival probability, while it was decreased in the oldest (80+ years) age group [**Figure 2A** (ii)]. In the older age groups (50+ years, majority of our cohort), increased risk of mortality was noted among Vax vs. NVax patients [**Figure 2A** (ii)]. Further, in our cohort, COVID-19 patients who received complete vaccination series had increased mortality risk compared to those received incomplete series (**Supplementary Figure 1A**). Additionally, we observed that Vax non-survivors had significantly increased average time between receiving the 1^st^ vaccine dose and hospital admission than surviving patients (**Supplementary Figure 1B**). Although the number of patients in this study is limited, these results suggest that among hospitalized patients, prior vaccination may not always be indicative of protection against mortality. One explanation of our findings might be that of hospitalized patients, Vax patients tend to be less healthy. In support of this, we found that Charlson’s Comorbidity Index score (CCI) was significantly higher in Vax vs. NVax (p=0.0002) patients [**Figure 2A** (iii)]. Additionally, age differed significantly between Vax (68 years) and NVax (62 years) patients [p=0.01, **Figure 2A** (iv)]. However, other factors are likely relevant and CCI or age may not be sufficient to stratify risk, as even when patients with similar CCI/age were compared, we still observed significantly (p=0.02) improved survival in NVax patients (**Figure 2B**). Importantly our results do not address the overall efficacy of vaccination with regards to mortality in a general population, as our analysis is limited to patients with severe infection admitted to the OSU hospital.

Our additional survival analyses demonstrated that the NVax patients admitted post-COVID-19 vaccine introduction had increased risk of mortality compared to those admitted pre-vaccine introduction (**Supplementary Figure 2A**). This could result from different dominant variants circulating during these different time periods (e.g. pre-Delta and Delta variants). However, Vax patients had significantly higher risk of mortality compared to both NVax groups (**Supplementary Figures 2B, C**).

In an attempt to control our observation, we assessed a cohort of 40 patients admitted to the hospital with non-COVID ARF. Mortality rates did not differ significantly between Vax (27%) and NVax (36%) patients (**Supplementary Table S1**). However, the number of patients in this cohort was also low, thereby limiting our ability to make conclusions.

**Figure 3:**
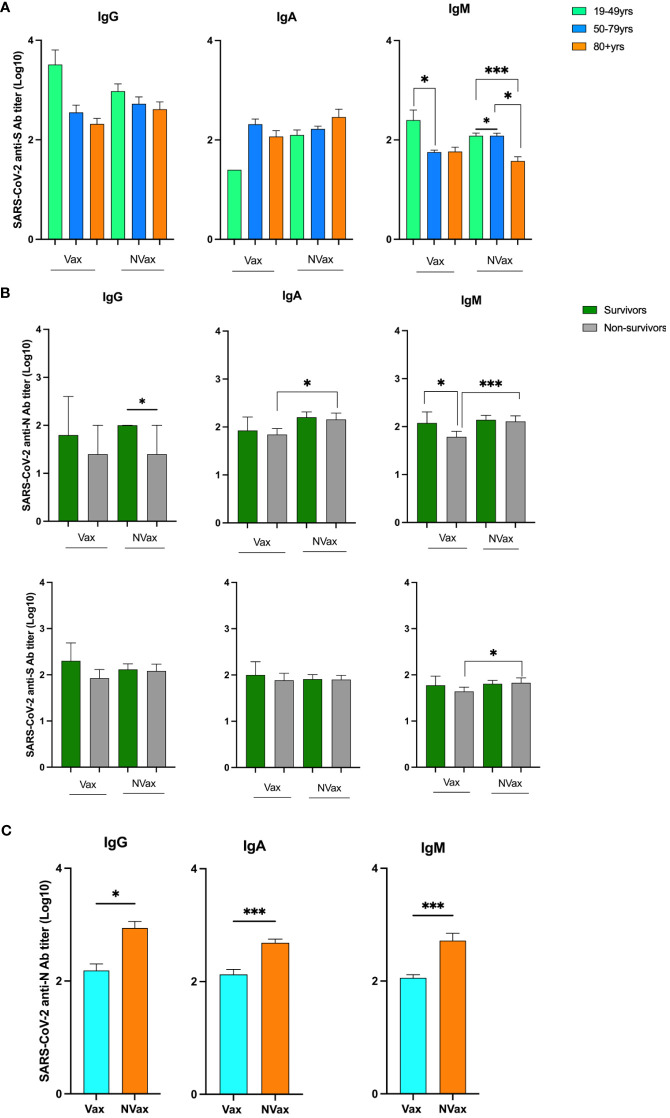
SARS-CoV-2 S and N peptide-specific IgG, IgA and IgM Ab titers in different age groups and surviving and deceased COVID-19 infected patients. SARS-CoV-2 N peptide-specific IgG, IgA and IgM Ab titers in the Vax and NVax SARS-CoV-2 infected patients. **(A)** SARS-CoV-2 anti-S Ab titers in Vax and NVax patients of different age groups; **(B)** SARS-CoV-2 anti-S and anti-N Ab titers in the Vax and NVax survivor vs. non-survivor patients; **(C)** SARS-CoV-2 anti-N Ab titers in the Vax vs. NVax patients. Differences were considered significant at a *p*-value < 0.05(*), and <0.001(***).

To evaluate the role of SARS-CoV-2/CCCoV-specific Abs in severe COVID-19 pathogenesis and immunity, we analyzed plasma levels of SARS-CoV-2/CCCoV IgG, IgA and IgM Abs of the SARS-CoV-2 infected patients. Assessment of Ab levels between these patient populations revealed some, but not definitive differences that stratified outcome. The SARS-CoV-2-S specific IgG/IgM Abs levels/titers of the younger Vax patients (19-49 years) were consistently higher than those of the NVax patients (**Figure 3A**). Further, non-survivors had lower SARS-CoV-2 N/CCCoV N Ab levels/titer compared to those patients who survived, and this trend was more pronounced in the Vax group (**Figures 3B** and **Supplementary Figure 3**). Additionally, the SARS-CoV-2-N specific IgG/IgA/IgM Abs titers of the NVax patients were significantly higher than those of the Vax patients (**Figure 3C**). Interestingly, NVax patients with comorbidities had higher SARS-CoV-2 specific Ab levels compared to their Vax counterparts (**Supplementary Figure 3**), suggesting that the presence of comorbidities was not the sole contributing factor to the decreased Ab response.

Thus, contrary to our expectations, we observed decreased Ab levels in the Vax compared to the NVax group. We next tested total IgG4 levels given emerging evidence that this isotype may have a role in the development of immunotolerance, and the association between COVID-19 mRNA vaccines and plasma IgG4 increases (7). Indeed, there was a trend for higher concentrations of total IgG4 Abs in the Vax vs NVax COVID-19 patients in week 3 (**Supplementary Figure 4**).

## Discussion

Vaccines have provided broad protection against COVID-19, with randomized trials in predominantly immunologically naïve individuals demonstrating high efficacy in preventing severe illness early in the pandemic (2). Surprisingly, in our limited dataset, COVID-19 vaccination was associated with worse clinical outcomes and decreased SARS-CoV-2/CCCoV Ab levels. Our data agree with previous studies reporting on increased in-hospital mortality rates in Vax vs. NVax patients with severe COVID-19 (5, 8–11). Of special relevance to our data (Supplementary Figure 1), Piotr Rzymski et al. reported that (9) among subgroups of Vax hospitalized patients (representing 1% of all hospitalized), mortality rates increased with additional vaccine doses and increased post-vaccination time (although deceased Vax patients represented only 0.2% of all hospitalized patients and 1% of all deceased individuals in the studied period). However, it is notable that other studies have reported benefit of prior vaccination for COVID-19 hospitalized patients (12–14). The reasons for these conflicting reports are not clear but it is possible that the timeframe of enrolment in our cohort may have resulted in the vaccinated population being more enriched for vulnerable individuals. However, there are multiple variables that may be relevant including the limited number of patients in our cohort.

Comorbidities and age are the known contributors to increased mortality among COVID-19 patients (5). Nonetheless, in our study, mortality remained significantly higher in the Vax patients even after adjustment for CCI, suggesting there are other risk factors in vaccinated patients.

This led us to investigate the immunological basis for our clinical observations. We observed reduced SARS-CoV-2-reactive Ab levels in Vax non-survivors. As a possible explanation for this observation, recent studies have shown that mRNA (but not vector-based) vaccine-associated increases in SARS-CoV-2 S-specific IgG4 levels vaccines did not contribute to increased protection (7, 8, 15). In contrast, they were thought to suppress antiviral immune responses, promoting immune tolerance and, possibly, unrestricted SARS-CoV-2 replication (7, 8, 16). In our study, the observed trend for increased total IgG4 concentration in week 3 for Vax patients may explain the reduced protective Ab responses. Of significance, clinical onset of IgG4-related non-infectious diseases is most often recorded in patients older than 50 years, which further corroborates our findings of increased mortality in this age group (16, 17).

Strengths: Our data suggest that comorbidities and advanced age are not the sole factors responsible for increased mortality risk among the SARS-CoV-2-infected Vax inpatients with ARF. There are several related possibilities that could explain this observation. One possibility is that patients at risk for severe outcome are more likely to receive vaccination and CCI does not fully stratify this risk. Similarly, it may be that a subset of Vax individuals have diminished protection for mechanisms not fully understood. Our observations suggest a potential role of IgG4 Ab mediated immune tolerance, but other mechanisms may be relevant, and our findings highlight the importance of further research.

## Limitations

While our investigation raises potentially important questions regarding the generalizability of vaccine efficacy, several limitations (including the small sample size) may impact the findings. Accordingly, these results require validation with further studies involving larger cohorts and must be interpreted cautiously. Further, the small sample size did not allow us to carefully evaluate the role of individual comorbidities [including immunosuppression, cancer, diabetes and pulmonary disease highly prevalent among the Vax patients (**Supplementary Table 1**)] that might influence Ab production. Additionally, the types and combinations of comorbidities varied between the Vax and NVax groups, which could contribute to the contrasting clinical outcomes. The timeframe of enrollment may also be relevant and contributed to more high-risk patients among our vaccinated cohort. Also, a longer observation period may be needed to identify significant trends in IgG4-response in Vax severe COVID-19 patients. As it is difficult to know the size of the population with prior vaccination or natural infection, and how these variables impact social risk factors related to risk of infection, it is not possible to make broad conclusions related to vaccine protection. Finally, our study population was limited to those patients hospitalized with severe infection, and studies have conclusively shown that vaccination significantly reduced the risk of hospitalization among the general population. Thus, our data may reflect the outcomes of a limited subset of patients with an altered host-response to vaccination.

## Conclusions

This study highlights the impact of vaccination, age, comorbidities, and Ab profiles on the clinical outcomes of COVID-19 ARF. While COVID-19 vaccination has played an important role in reducing COVID-19-related hospitalization risk, the observation of higher mortality rates among 50+ Vax vs. NVAx patients with severe COVID-19 infection and respiratory failure is a matter of concern. This finding raises the possibility that an aberrant immunopathological response to vaccination in a subset of patients might have contributed to the inadequate response to COVID-19 vaccines. This speculative observation warrants additional research regarding heterogeneous host responses to vaccination. It is also important to note that overall frailty is recognized as a reliable predictor of clinical outcomes of COVID-19 followed by age and comorbidities. However, elderly, and those with various comorbid conditions and increased frailty index, were not adequately represented in the conducted clinical COVID-19 vaccine trials. This significant omission is highlighted by our current and other similar studies and suggest the need for better vaccines or other therapies for certain at-risk populations.

## Data availability statement

The original contributions presented in the study are included in the article/**Supplementary Material**. Further inquiries can be directed to the corresponding author.

## Ethics statement

The studies involving humans were approved by Ohio State Biomedical Sciences IRB (IRB # 2020H0198) of the Ohio State University. Samples were obtained from the Ohio State University Intensive Care Unit Registry (BuckICU, IRB approval #2020H0175) biorepository. The studies were conducted in accordance with the local legislation and institutional requirements. The participants provided their written informed consent to participate in this study.

## Author contributions

BA: Data curation, Formal analysis, Investigation, Methodology, Software, Validation, Writing – original draft, Writing – review & editing. JB: Resources, Writing – review & editing. JH: Resources, Writing – review & editing. MR: Writing – review & editing. AV: Conceptualization, Data curation, Formal analysis, Methodology, Project administration, Software, Supervision, Validation, Visualization, Writing – original draft, Writing – review & editing.

## Supplementary material

Supplementary Figure 1 Overall survival probability of the Vax (incomplete and complete vax series) and NVax SARS-CoV-2-infected patients **(A)** and average time post-first vaccine dose – to hospitalization among Vax survivors and non-survivors **(B)**.

Supplementary Figure 2 Overall survival probability of the NVax patients admitted pre- and post-COVID-19 vaccine approval **(A)**, of the Vax patients vs. NVax patients admitted pre-COVID-19 vaccine approval **(B)**, and of the Vax patients vs. NVax patients admitted post-COVID-19 vaccine approval **(B)**.

Supplementary Figure 3 CCCoV and SARS-CoV-2 S and N peptide-specific IgG, IgA and IgM Ab levels (presented as OD_650_ values) in Vax and NVax SARS-CoV-2 infected patients analyzed based on the comorbidity **(A)** [because 100% of the Vax patients had comorbidities, we compared them to the NVax patients with comorbidities, excluding those without], patient age **(B)**, and survivor vs. non-survivor status **(C)**.

Supplementary Figure 4 Total IgG4 concentrations in week 1, week 2, and week 3 of SARS-CoV-2 infected Vax vs. NVax patients.

Supplementary Table 1 Baseline clinical characteristics and outcomes (n. (%) or median). *Green color indicates statistically significant differences.
