# Corrigendum: Brief research report: impact of vaccination on antibody responses and mortality from severe COVID-19

**DOI:** 10.3389/fimmu.2024.1384209

**Published:** 2024-02-28

**Authors:** Bindu Adhikari, Joseph S. Bednash, Jeffrey C. Horowitz, Mark P. Rubinstein, Anastasia N. Vlasova

**Affiliations:** ^1^ Department of Veterinary Preventive Medicine, College of Veterinary Medicine, The Ohio State University, Wooster, OH, United States; ^2^ Center for Food Animal Health, Department of Animal Sciences, Ohio Agriculture Research and Development Center (OARDC), College of Food, Agricultural and Environmental Sciences, The Ohio State University, Wooster, OH, United States; ^3^ Department of Internal Medicine, Division of Pulmonary, Critical Care, and Sleep Medicine, The Ohio State University, Columbus, OH, United States; ^4^ Division of Medical Oncology, Department of Internal Medicine, The Ohio State University, Columbus, OH, United States; ^5^ The Pelotonia Institute of Immuno-Oncology, The Ohio State University James Comprehensive Cancer Center, Columbus, OH, United States

**Keywords:** SARS-CoV-2, mortality, antibodies, IgG4, immune tolerance, comorbidities, COVID-19 vaccines

In the published article, there was an error. The authors cited a previous publication in a way that can be misinterpreted if taken out of context.

A correction has been made to **Discussion**, paragraph 1, page 4.

This sentence previously stated:

“Of special relevance to our data (Supplementary Figure 1), Piotr Rzymski et al. reported that (9), counterintuitively, mortality rates increased with additional vaccine doses and increased postvaccination time”.

The corrected sentence appears below:

“Of special relevance to our data (Supplementary Figure 1), Piotr Rzymski et al. reported that (9) among subgroups of Vax hospitalized patients (representing 1% of all hospitalized), mortality rates increased with additional vaccine doses and increased post-vaccination time (although deceased Vax patients represented only 0.2% of all hospitalized patients and 1% of all deceased individuals in the studied period).”

The authors apologize for this error and state that this does not change the scientific conclusions of the article in any way. The original article has been updated.

