# Supplementary material for: Brief research report: impact of vaccination on antibody responses and mortality from severe COVID-19
Source: Front Immunol. 2024 Feb 7;15:1325243. doi: 10.3389/fimmu.2024.1325243 (PMC10883056; doi:10.3389/fimmu.2024.1325243)
Supplement: Supplementary Figure 1 — Overall survival probability of the Vax (incomplete and complete vax series) and NVax SARS-CoV-2-infected patients (A) and average time post-first vaccine dose – to hospitalization among Vax survivors and non-survivors (B). [file DataSheet_1.pdf]

Supplemental data

Supplemental Table 1.

Baseline clinical characteristics and outcomes (n. (%) or median)

	All<br>n=112	NVax<br>n=89	Vax<br>n=23	p-value
**SARS-CoV-2 infected**				
Age	64	62	68	0.011*
Male	59(53%)	49 (55%)	10 (43%)	0.356
Heart disease	92 (82%)	71 (80%)	21 (91%)	0.239
Pulmonary disease	45 (40%)	32 (36%)	13 (57%)	0.095
Liver disease	7 (6%)	4 (5%)	3 (13%)	0.151
Diabetes	48 (43%)	34 (38%)	14 (61%)	0.06
Hematological disorder	2 (2%)	2 (2%)	0 (0%)	0.99
Immunosuppression	26 (23%)	15 (17%)	11 (48%)	0.004*
Cancer	26 (23%)	17 (19%)	9 (39%)	0.054
Mortality	49 (44%)	33 (37%)	16 (70%)	0.0086*

**Non-infected**	All<br>n=40	NVax<br>n=25	Vax<br>N=15	p-value
Age	65	66	64	0.98
Male	22 (55%)	13 (52%)	9 (60%)	0.747
Heart disease	31 (78%)	20 (80%)	11 (73%)	0.705
Pulmonary disease	15 (38%)	10 (40%)	5 (33%)	0.745
Liver disease	3 (8%)	3 (12%)	0 (0%)	0.278
Diabetes	14 (35%)	10 (71%)	4 (29%)	0.502
Hematological disorder	2 (5%)	2 (8%)	0 (0%)	0.519
Immunosuppression	7 (18%)	5 (20%)	2 (13%)	0.691
Cancer	13 (33%)	4 (31%)	9 (69%)	0.0063*
Mortality	13 (33%)	9 (36%)	4 (27%)	0.730

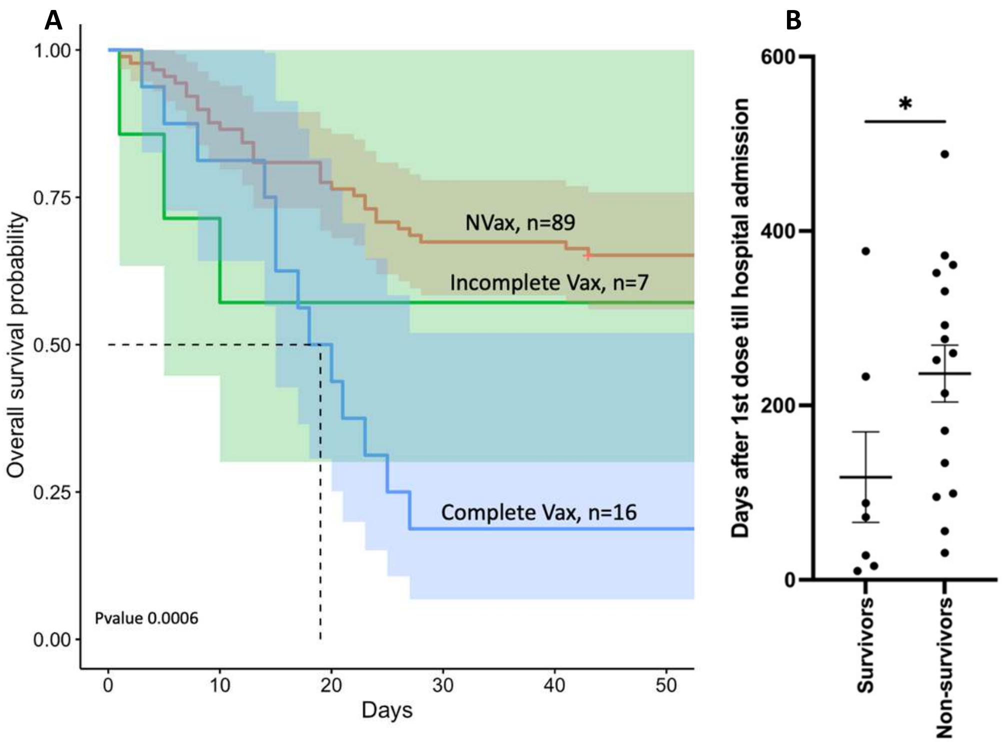

Supplemental figure 1. Overall survival probability of the Vax (incomplete and complete vax series) and NVax SARS-CoV-2-infected patients (A) and average time post-first vaccine dose to hospitalization among Vax survivors and non-survivors (B).

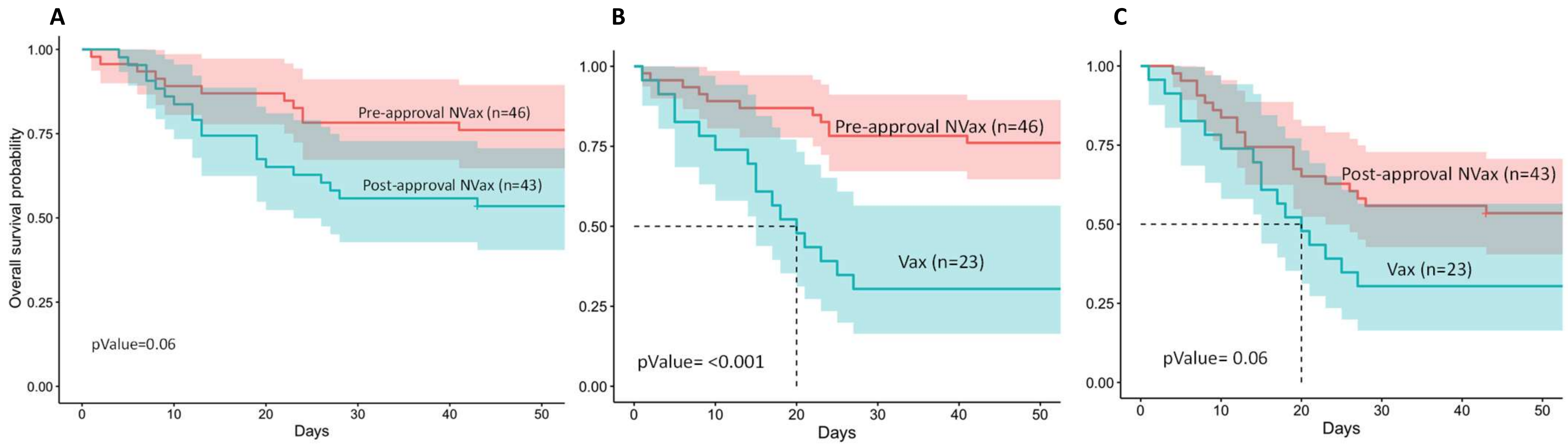

Supplemental figure 2. Overall survival probability of the NVax patients admitted pre- and post-COVID-19 vaccine approval (A), of the Vax patients vs. NVax patients admitted pre-COVID-19 vaccine approval (B), and of the Vax patients vs. NVax patients admitted post-COVID-19 vaccine approval (B).

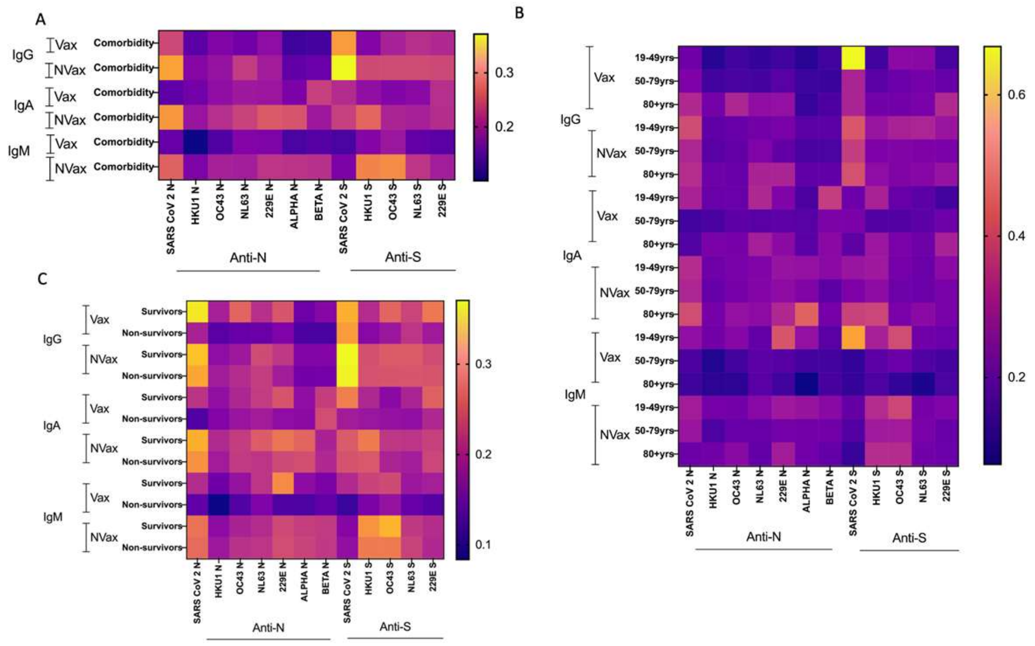

Supplemental figure 3. CCCoV and SARS-CoV-2 S and N peptide-specific IgG, IgA and IgM Ab levels (presented as OD_{650} values) in Vax and NVax SARS-CoV-2 infected patients analyzed based on the comorbidity (A) [because 100% of the Vax patients had comorbidities, we compared them to the NVax patients with comorbidities, excluding those without], patient age (B), and survivor vs. non-survivor status (C).

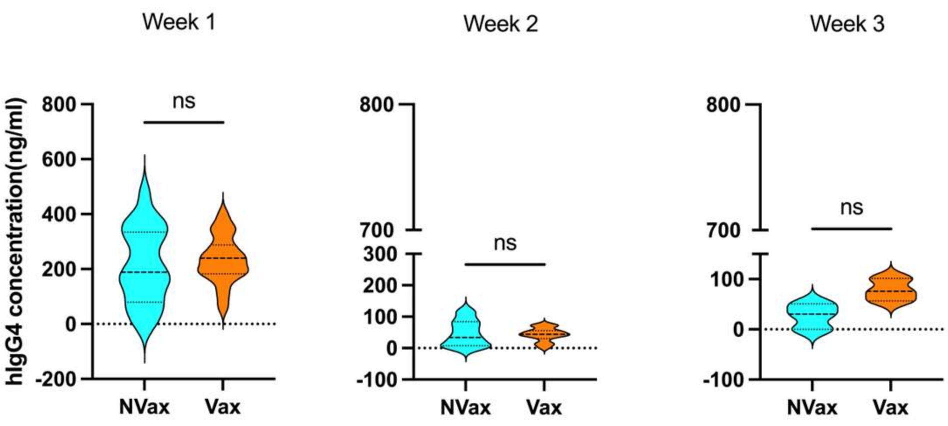

Supplemental figure 4. Total IgG4 concentrations in week 1, week 2, and week 3 of SARS-CoV-2 infected Vax vs. NVax patients.
